# Conflict and control in cortical responses to inconsistent emotional signals in a face-word Stroop

**DOI:** 10.3389/fnhum.2023.955171

**Published:** 2023-06-30

**Authors:** Graham A. Jamieson, Julia Page, Ian D. Evans, Adam Hamlin

**Affiliations:** ^1^School of Psychology, University of New England, Armidale, NSW, Australia; ^2^School of Science and Technology, University of New England, Armidale, NSW, Australia; ^3^Illawarra Health and Medical Research Institute, University of Wollongong, Wollongong, NSW, Australia

**Keywords:** affective Stroop, N170, amygdala, rostral ACC, fusiform face area, affective control, eLORETA

## Abstract

Social communication is fraught with ambiguity. Negotiating the social world requires interpreting the affective signals we receive and often selecting between channels of conflicting affective information. The affective face-word Stroop (AFWS) provides an experimental paradigm which may identify cognitive-affective control mechanisms underpinning essential social-affective skills. Initial functional magnetic resonance imaging (fMRI) study of the AFWS identified right amygdala as driving this affective conflict and left rostral anterior cingulate cortex (rACC) as the locus of conflict control. We employed electroencephalogram (EEG) and eLORETA source localization to investigate the timing, location, and sequence of control processes when responding to affective conflict generated during the AFWS. However we designated affective word as the response target and affective face as the distractor to maximize conflict and control effects. Reaction times showed slowed responses in high vs. low control conditions, corresponding to a Rabbitt type control effect rather than the previously observed Grattan effect. Control related activation occurred in right rACC 96–118 ms post-stimulus, corresponding to the resolution of the P1 peak in the Visual Evoked Potential (VEP). Face distractors elicit right hemisphere control, while word distractors elicit left hemisphere control. Low control trials require rapid “booting up” control resources observable through VEPs. Incongruent trial activity in right fusiform face area is suppressed 118–156 ms post stimulus corresponding to onset and development of the N170 VEP component. Results are consistent with a predicted sequence of rapid early amygdala activation by affective conflict, then rACC inhibition of amygdala decreasing facilitation of affective face processing (however, amygdala activity is not observable with EEG).

## 1. Introduction

Negotiating the complex, often contradictory world of social communication requires skillful understanding not only of the goals, thoughts, and intentions of others, but also of their emotions expressed across both verbal and non-verbal channels of communication. Due to the pragmatic nature of social communication, emotional signals may be both consistent and inconsistent across verbal and non-verbal channels, and this may change rapidly across time.

In turn, the perceived emotions of others often evoke corresponding emotions, which play a powerful role in motivating and regulating our own social responses (Ratcliffe, 2018). Consequently, an understanding of how the brain responds to such changes in consistency and inconsistency between verbal and non-verbal emotional signals is an important task for a humanly meaningful social and affective neuroscience.

Early cognitive neuroscience research leveraged a range of experimental paradigms taken from experimental psychology which elicited objective behavioral effects (reaction time and accuracy) in response to various manipulations of task conflict in order to identify and parse the neural responses underlying these well-known behavioral effects. These paradigms pitch various aspects of the experimental stimulus (e.g., location, features, context), selection of response category, motor response (e.g., right or left hand), and task requirements (e.g., name the word or name the color) to create conflicts affecting selection of the required behavioral response with observable effects on reaction time and accuracy. For example, the color-naming Stroop paradigm requires participants to respond to the *color* of the word while ignoring the written word, which may be congruent (e.g., when the word BLUE is presented in a blue color) or incongruent (e.g., when the word BLUE is presented in a yellow color) with the required behavioral response. Similarly, the Eriksen Flanker task requires participants to respond to the central letter or symbol in a row of symbols, where it is flanked by characters which may be congruent or incongruent with the required behavioral response (Egner, 2008).

Then as now, theories of top-down regulation (control) posit that processing conflicts elicited by these paradigms mobilize resources which regulate these conflicts in the service of task performance (Botvinick et al., 2004; Carter and van Veen, 2007). Thus, neuroimaging studies have employed these paradigms to identify both the locus of specific processing conflict effects and of the control responses theorized to accompany them. An important limitation of these studies is the difficulty in establishing the time course of these interacting neural processes due to the temporal resolution of most brain imaging technologies. This information is critical not only for the testing of specific theoretical models, but also for the development of alternative theoretical models. One way in which this can be addressed is by employing source localization of the electroencephalogram (EEG), with high temporal resolution, to specify the timing of local functional responses identified in brain imaging research. This is the approach adopted in the present study, leveraging first generation brain imaging affective neuroscience findings, to identify both the locus and timing of affective processing conflict and the control of affective conflict engendered by conflicting verbal (word) and non-verbal (facial expression) affective stimuli in a Stroop type paradigm (Etkin et al., 2006).

One approach taken to identify the locus of conflict processing effects in the Stroop or related paradigms is to identify brain activity following the presentation of stimuli combining task relevant and irrelevant features with conflicting task response mappings (incongruent or I stimuli/trials), and contrast that with brain activity following stimuli combining such features but with identical task response mappings (congruent or C stimuli/trials; e.g., MacDonald et al., 2000). The classic Stroop conflict effect is caused by I trials producing high levels of conflict while C trials produce relatively low levels of conflict, resulting in slower RTs in I rather than C trials. In the case of Stroop type paradigms, processing conflict is elicited between competing and exclusive behavioral responses, but might in principle also occur *earlier* at the level of stimulus processing (which underscores the importance of determining the timing of such effects). However, I trials are also expected to elicit activation of control resources to maintain the accuracy of responses in the face of Stroop type response conflicts, whereas C trials do not. Taken alone, regions of increased activation in the I vs. C comparison could indicate either or both the effects of task conflict (during stimulus or response processing) or the control processes (at the locus of either their origin or their expression) elicited to regulate this conflict.

Botvinick et al. (1999) devised a useful experimental logic based on the Gratton effect (Gratton et al., 1992) to parse cognitive control from conflict effects in neuroimaging studies of experimental conflict paradigms. Gratton et al. (1992), employing a version of the Eriksen Flanker task, reported that current trial processing conflict (as indexed by RT and accuracy) was sensitive to prior trial congruency with increased accuracy and decreased RT on high conflict I trials when they were preceded by an I trial (II trial sequence) than when preceded by a C trial (CI trial sequence). For low conflict C stimuli, this relationship was reversed such that RT increases and accuracy decreases when preceded by a I stimulus (IC trial sequence) than when preceded by a C stimulus (CC trial sequence). This effect is interpreted as due to prior activation of control resources on high conflict I trials (which is absent on prior C trials). Thus, II trials are expected to be higher in control and lower in processing conflict than CI trials. Following this logic, activation in brain regions which is greater in CI than II trials may be interpreted as due to processing conflicts and brain activation which is greater in II than CI trials due to control processes, thereby dissociating conflict effects from control effects (which are conflated in a direct I vs. C contrast).

This logic was employed by Botvinick et al. (1999) to determine that anterior cingulate cortex (ACC) activation in the Flanker paradigm was sensitive to processing conflict (CI > II) rather than control processing, a critical prediction of the theory of conflict monitoring and cognitive control (Botvinick et al., 2001). Egner and Hirsch (2005a) applied this same logic to identify brain regions which either implement or express cognitive control (CI < II) in response to processing conflict on the color-word naming Stroop task, finding control specific activation in left midfrontal and superior frontal gyrus. In order to demonstrate the effects of control specific activation on task specific sensory processing, Egner and Hirsch (2005b) developed a novel Stroop type task using face-word stimuli, where the task was to identify either the word or the face as that of a famous actor or famous politician. Face stimuli were chosen to examine the effects of control related activation on activity in target specific sensory processing pathways (i.e., on the expression of control). The logic of this design utilizes activation in the fusiform face area (FFA), an extrastriate visual area specific to the processing of face stimuli (Kanwisher and Yovel, 2006). Egner and Hirsch (2005b) determined that when faces served as targets, activity in FFA was significantly greater in the high control (II) than low control (CI) conditions. That is, the FFA was found to be the site for the expression (as distinct from the source) of conflict control effects in this context. Furthermore, functional connectivity between the FFA and control related activity in right dorsolateral prefrontal cortex (CI < II) was also enhanced in high vs. low control conditions during for face as target responses consistent with the latter region acting as the source of control related modulation expressed in the former. This paradigm became the basis for the development of the AFWS employed by Etkin et al. (2006) to study the control of interference caused by conflicting affective signals.

Both everyday experience and experimental studies point to the tendency for strong emotional experiences (and the situations which elicit them) to interfere with the speed and accuracy of behavior. Such interference between affect and cognition has been investigated using “emotional Stroop” paradigms, in which affectively arousing stimuli are presented in conjunction with (or incorporated within) stimuli to be processed in some cognitive paradigm. Affective stimuli employed in such paradigms are normatively graded according to valence and intensity (Sutton et al., 2019) with both dimensions exerting distinct interference effects with attention to ongoing “cold” cognitive processing (Sutton and Lutz, 2019). It should be noted that this is not the type of affective interference targeted by Etkin et al. in the design of their AFWS task. Rather, they sought to examine the conflict created between mutually exclusive affective signals and the neural processes elicited to control such conflict.

Face-word Stroop paradigms present visual stimuli where a word is superimposed upon a face, and participants respond to either the word or the face by identifying a salient stimulus dimension of the target feature such as whether the target is an actor or a politician (Egner and Hirsch, 2005b) or whether the target is male or female (Egner et al., 2010). Face and word pairings may be matching (congruent: C) or conflicting (incongruent: I) with respect to the target dimension. In Stroop-type paradigms, responses to incongruent trials are slower than responses to congruent trials (MacLeod, 1991) and many brain imaging studies have employed such paradigms in the cognitive domain to study neural processes underlying response conflict and the resolution of response conflicts within the brain. By presenting trials in a mixed sequence, Egner et al. (2008) sought to leverage their analysis of underlying processes by taking advantage of methods applied in previous brain imaging studies of cognitive control using Stroop type conflict paradigms (Kerns et al., 2004). Etkin et al. (2006) applied this methodology to the study of emotional conflict processing by pairing an affectively expressive face (happy or fearful) with the overlaid word “happy” or “fear” to produce a direct analogue of the perceptual, semantic, and response conflicts characteristic of cognitive Stroop type tasks in the affective domain. Due to the central importance of those findings to the current study we will next consider them in some detail.

At a behavioral level, Etkin et al. (2006) found both Stroop and Gratton effects. For congruent vs. incongruent trials, they carried out a region of interest contrast on the amygdalae which found increased activation in the right amygdala for all incongruent trials, concluding that the amygdala is responsive to conflict in emotional information. For the control related contrast of II vs. CI trials, analyses were conducted on regions of interest in bilateral dACC and medial prefrontal cortex, lateral prefrontal cortex, and rostral anterior cingulate cortex (rACC). CI trials resulted in greater activation than II trials in bilateral dACC, medial prefrontal cortex, and lateral prefrontal cortex. This was interpreted as conflict (rather than control) related, as the Gratton effect was taken to indicate that conflict was higher in CI than II trials due to the slower reaction times (RTs) of the former.

II trials (compared to CI) produced greater activation in the left rACC, which was interpreted as control related effects (based on faster RTs to II than CI trials). This interpretation was bolstered by a series of further analyses; activity in medial PFC and right DLPFC during prior incongruent trials was found to predict rACC activation in the current trial. Psychophysiological interaction analysis (Friston et al., 1997) found an inverse relationship between current trial activity for rACC and amygdala for high control (II) but not low control (CI) trials. The extent to which left rACC activity predicted reduced right amygdala activity in II vs. CI trials was also found to predict the extent to which RT on II trials was reduced in comparison to CI trials (a.k.a., the Gratton effect). An effective connectivity analysis (Friston et al., 2003) confirmed that prior trial incongruence enhanced rACC negative effective connectivity (inhibitory control) over the amygdala on the following trial. No rACC effects were found in the contrast between high and low conflict (I vs. C) trials. Etkin et al. (2006) therefore concluded that the rACC plays a key role in the resolution of processing conflicting affective signals, at least in part by the regulation of the affective (including autonomic) responses to those mixed verbal and non-verbal signals.

Etkin et al. (2006) were able to identify context dependent contralateral relations between the amygdala and rACC related to control within trials, and corresponding dynamic relations between them across trials. However, their account logically predicts a specific (unfolding) sequence of within-trial effects that is beyond the temporal resolution of standard fMRI methods to identify. Firstly, rACC control related activation should follow activation of the amygdala by conflicting affective visual stimuli. Secondly, if rACC exerts control of affective conflict by downregulating this amygdala response, it should precede a reduction in later activation during the processing of conflicting affective stimuli that would otherwise be facilitated by this initial amygdala activation. The timing of these relationships cannot be established within the temporal resolution of fMRI but may be accessible through the evoked cortical responses generated by conflicting visual affective stimuli.

The amygdalae (left and right) are amongst the most omniconnected regions in the brain (Pessoa, 2008). These connections include subcortical regions regulating sympathetic nervous system reactivity (Beissner et al., 2013), hippocampal regions involved in the memory of reward contingencies (Yang and Wang, 2017; Murray and Fellows, 2022), frontal cortex including rACC and orbitofrontal cortex (Beckmann et al., 2009), and all levels of visual processing from striate to extrastriate cortex. Thus, the amygdalae (left and right) are principal hubs in the integration affective and cognitive responses to perceptual signals within the brain.

Initial activation of the amygdala by affectively salient facial expressions (fear and happiness) occurs rapidly via magnocellular pathways from the superior colliculus and pulvinar nucleus of the thalamus (Garvert et al., 2014). Synaptic transmission time along this pathway is estimated to take 80–90 ms (McFadyen et al., 2019). Detailed visual processing of these same stimuli occurs later along a pathway which feeds forwards from specific feature identification in the occipital face area (Pitcher et al., 2011) to feature integration into facial gestalts in inferior temporal cortex and the FFA (Ishai, 2008). Anatomical studies in primates demonstrate that the amygdala projects extensively to both earlier and later regions activated in this visual processing hierarchy (Adolphs and Spezio, 2006) but most strongly to later processing regions of the fusiform gyrus (Pessoa, 2008). Morris et al. (1998) and Garvert et al. (2014) have provided evidence for top-down amygdala modulation of the processing of facial expressions in occipital and temporal visual cortex, respectively.

Based on these findings, it is expected that amygdala activation triggered by processing conflicts between incongruent sensory affective signals in the affective face-word Stroop task will drive the amplification of conflicting representations of affective meaning generated at higher levels of visual (face and word) processing hierarchies. In which case, the down-regulation of conflict-related amygdala activation by rACC reported by Etkin et al. (2006) would be expected to lead to decreased facilitation of the visual processing pathway engaged by task incongruent affective information. Although both face and word present affectively valenced visual information, affective word recognition is expected to rely upon a left hemisphere occipital to temporal visual processing pathway while affective face recognition will depend upon a right hemisphere occipital to temporal visual pathway. It may be noteworthy that control-related rACC activation in that study was only observed in the left rACC—the same hemisphere in which the conflicting affective signal (the word) is processed.

We considered that application of EEG source localization methods to the averaged visual evoked potentials (VEPs) generated by a version of face-word affective Stroop may permit us to test aspects of the functional interpretation developed above. VEPs provide the fine-grained temporal resolution to allow us to establish the timing and temporal sequence of functional events with great precision. Source localization, guided by prior results and specific predictions, permits us to identify whether the cortical regions expected to be engaged in those events match the actual regions involved.

The features of the VEP of interest for the current study are early waveforms generated by occipital and temporal cortex, which are specifically responsive to face processing. These are the P1 wave (typically arising about 60 ms post-stimulus and reaching a peak about 100 ms post-stimulus) and the immediately following N170 wave peaking about 150–170 ms post-stimulus (Luck, 2014). There is a voluminous literature on both waveforms in the event related potential literature which we do not seek to address here. What is important for the current study is that the P1 is the earliest waveform in the VEP sensitive to face related stimulus features (Pitcher et al., 2011) while the N170 is associated with numerous forms of whole face discrimination, including the affect of facial expressions (Alguacil et al., 2017; Qiu et al., 2017). Generators contributing to the P1 (in the context of face stimuli) include lateral and mid occipital cortex (Di Russo et al., 2002; Pitcher et al., 2011). While the contributions of source activity in the FFA to the N170 have been a matter of controversy (Eimer, 2011; Luck, 2012), a recent direct electro-cortical recording study shows that face elicited activity in the right FFA coincides with the timing of the scalp recorded N170 (Jacques et al., 2019). Another recent study using individual structural MRI images as head models for cortical source analysis of the N170 reported that the FFA is the major contributor to the face-sensitive N170 (Gao et al., 2019).

**FIGURE 1:**
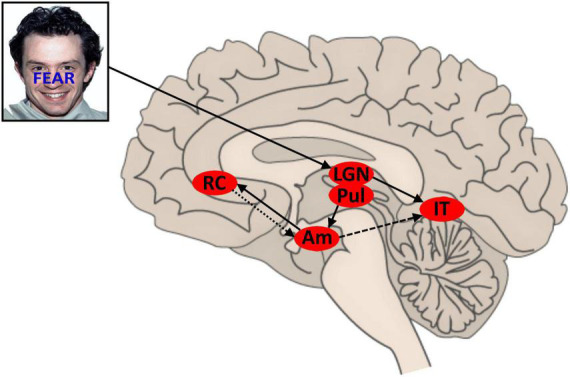
Proposed model for conflict and control in AFWS word-task. Face stimuli sourced from the NimStim set of Facial Expressions (Tottenham et al., 2009) and available to the scientific community at https://macbrain.org/resources/. All faces are from the Nim Stim data set publicly available for scientific research. LGN, lateral geniculate nucleus; Pul, pulvinar nucleus; AM, amygdala; IT, inferior temporal region; rACC, rostral anterior cingulate cortex (BA32); solid line, feedforward signal; light dash, inhibition; heavy dash, (variable) facilitation.

A tentative model of the temporal dynamics in control responses to affective stimulus conflict may be drawn: initial conflicting affective signals are relayed rapidly to the amygdalae. Conflict sensitive (possibly lateralized) amygdala activation triggers a rapid inhibitory control response from (possibly contralateral) BA32 and enhances processing of affective face features in (likely right) inferior temporal lobe (associated with the FFA). This facilitation is downregulated as a consequence of BA32 regulation of the amygdala in a later time window of the VEP (see Figure 1). The likely time course of amygdala activation and down regulation will be reflected in aspects of sympathetic nervous system activity, but that is beyond the scope of this paper.

We set out to test predictions drawn from this model which extend the temporal detail reported by Etkin et al. (2006) for the AFWS task. In particular, we set out to establish the timing of BA32 control activation elicited by conflicting affective stimuli and to establish the relationship of that timing relative to the expression of control in the sensory processing of conflicting affective stimulus features. We recorded scalp EEG while administering a version of the AFWS paradigm and later conducted eLORETA source analysis (Pascual-Marqui et al., 2011) on the obtained VEPs in time windows corresponding to the rise and fall of the P1 and N170 components. Following Etkin et al., we compared I vs. C trials to identify the timing and expression of conflict control and II vs. CI trials in order to determine the timing of predicted rACC affective-conflict control. Scalp recorded EEG is unable to detect electrical activity generated by the amygdala.

We chose to depart from the paradigm employed by Etkin et al. in one very important respect: while they employed the face as the response target, we employed the word. In the results of the initial (non-affective) face-word paradigm reported by Egner and Hirsch (2005b), the statistical magnitude of the conflict effect (C vs. I reaction time difference) is much larger for the word target that the face target task. Similarly, Ovaysikia et al. (2011) compared responses to emotion word and affective faces in a similar affective face-word Stroop experiment and reported the statistical magnitude of the classic Stroop effect for RT (I > C) to be greater for the word target compared to the face target. Due to the central role of conflict in eliciting affective control in this paradigm, it was decided to adopt a word target version of the task in order to maximize the magnitude of the affective control response in the present study. In this case, further processing of the affective face stimulus becomes the source of task interference. Additionally, both the timing of VEP components and the location of the extrastriate processing pathways engaged in affective face processing are known in much greater detail than for affective words.

The issues addressed in the current study (together with the methods of inquiry and analysis) are driven by unanswered questions arising from the rationale, findings and conclusions lying in and behind the original paper of Etkin et al. (2006). While there have been over 1,600 published citations of that paper to date, to our knowledge the simple questions posed here (originating directly from that original paper) have not been addressed in any of those studies. There is, however, a group of studies likely to be of particular relevance to those which employ neuroscience methods to study word target versions of the AFWS.

Zhu et al. (2010) conducted a classical ERP study comparing VEPs to congruent and incongruent AFWS stimuli for both word target and face target versions of the task. They sought to demonstrate affective face processing effects in the early N170 component of the VEP. When responding to the face, I-trials evoked a more negative N170 compared to C trials. Alternately, responding to the word evoked a *less* negative N170 in I-trials. This indicates that the N170 is indeed sensitive to conflict in affective stimulus, and that modulation (control) of task relevant and task irrelevant affective stimulus features have been triggered by this very early processing stage, supporting our choice to examine these early time windows. Behavioral findings from the fMRI study by Ovaysikia et al. (2011) have been reviewed above, supporting our choice of word target in the current paradigm. That study also reports a comparison of the BOLD response between I and C trials in word and face target conditions respectively finding both overlap and differences in regions active during conflict for the word and face target. Perhaps most salient to the current study, conflict (i.e., I > C) showed greater activation in right inferior temporal regions for the word but not the face target. This is consistent with enhanced processing of affective facial features triggered by conflict when the face acts as distractor, but the precise timing of such an event cannot be determined from this study.

Yang et al. (2016) also report an fMRI study using a word target version of the AFWS. They conducted separate contrasts for negative and positive affective face stimuli (distractors) to determine regions engaged in conflict effects in each case. Significant conflict effects were observed for positive but not for negative affective faces. Valence of the affective face was a major factor in determining conflict effects. The impact of the valence of affective faces on conflict control, the focus of the current study, remains to be determined. It is clearly an important and open question whether or not control responses to affective conflict (II > CI) are linked to a specific valence of the affective face however we chose not to pursue that question in the current study.

Following these considerations, we sought to test the following predictions:

1. We predicted that, when identifying the word of the affective face-word Stroop task, participants would still show the classic Stroop effect; that is, faster reaction times to congruent face-word affective signals than to incongruent signals.
2.
  a) We also expected conflict related control effects in the form of significant reaction time differences between II and CI trial types.
  b) We expected these differences to follow the Gratton effect with reaction time for II trials faster than for CI trials.
3. We sought to employ EEG source localization of VEP components to determine the timing of rACC affective-conflict related control activity as reported by Etkin et al. (2006).
  a) We expected eLORETA estimates of source localized activity during the VEP to face-word stimuli, in the rACC, to be significantly greater in II than CI trials.
  b) Due to the reversal in the laterality of stimulus processing for the incongruent distractor (from word to face, from left hemisphere to right hemisphere) we expected rACC control activation to be switched from left rACC (in Etkin et al., 2006) to right rACC in the present study.
  c) We expected control related rACC activation to occur in a time window following expected amygdala activation (80 ms) but prior to control induced downregulation of (conflicting) affective-face processing.
4. We propose that affective face-word conflict triggers a rapid control response that for word targets leads to reduced facilitation of the affective component of face processing. Therefore, we expected a reduction in activity in the right FFA in the time window of the N170 for I trials compared to C trials.

## 2. Materials and methods

### 2.1. Participants

Forty-eight English speaking employees (*Male* = 19, *Female* = 29) from a local government authority at a regional Australian city volunteered for this study. Ages ranged from 25 to 65 years (M = 46.3, SD = 12.3). A total of 38 participants self-reported right-handedness and 10 self-reported left-handedness. Eight participants declared they were taking prescription medication for either a neurological or psychological condition. Ethics approval was granted by the responsible Human Research Ethics Committee. All participants provided written informed consent prior to the experiment and were debriefed on completion of their testing. Permission to conduct the study was granted by the local government authority’s Chief People and Culture Officer.

### 2.2. Experimental paradigm

**FIGURE 2:**
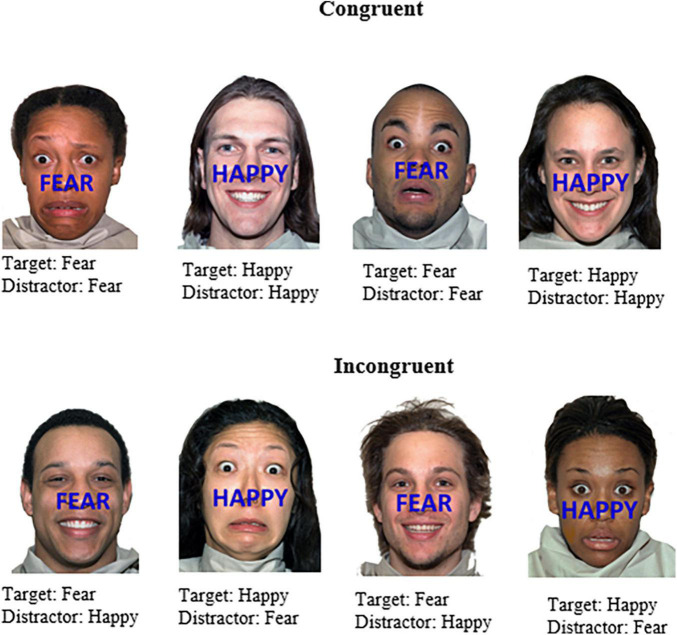
Example stimuli of the affective face-word Stroop task. Stimuli were either congruent with respect to the word (emotion target) and face (emotion distractor) or incongruent, which were designed to elicit affective conflict. Participants were instructed to identify the emotion word that appeared across the face (i.e., happy or fear). Face stimuli sourced from the NimStim set of Facial Expressions (Tottenham et al., 2009) and available to the scientific community at https://macbrain.org/resources/. All faces are from the Nim Stim data set publicly available for scientific research.

An adaption of the emotional face-word Stroop task (Etkin et al., 2006) was created and employed to induce emotional conflict arising from the incongruence between two emotional dimensions of a stimulus. Photos of faces with fearful or happy expressions constituted the task-irrelevant dimension of the stimulus, whereas the task-relevant dimension involved identifying the emotional word that appeared across the face. Stimuli were presented with STIM2 software (Compumedics USA Ltd, El Paso, TX, USA) on a 51 cm LCD monitor. The task consisted of happy and fearful faces selected from The Research Network on Early Experience and Brain Development’s battery of 646 facial expression stimuli, developed for use in studies of face and emotion recognition (Tottenham et al., 2009). All faces in that data set are of paid actors. Images were matched on brightness, contrast, and size. Fearful and happy faces were chosen to elicit strong emotional/somatic responses. The faces were presented in color and the words “FEAR” or “HAPPY” appeared in blue capital letters 2 cm tall on screen and were positioned centrally across the face above the top lip across the nose. The facial expression and word were either congruent or incongruent (refer Figure 2 for details).

Four different trial types were created on this combination of facial expression and emotional word, i.e., 43 congruent-incongruent (CI), 51 incongruent-incongruent (II), 44 incongruent-congruent (IC), and 14 congruent-congruent (CC). As the planned analyses was the CI vs. II comparison (with C vs. I as a secondary analysis) the stimulus sequence was constructed to maximize the occurrence of CI and II trial types. CC and IC trial types as such are not analyzed. The number of fearful/happy faces for each relevant condition can be seen in Table 1. All experimental stimuli and trial sequences employed here may be viewed or downloaded from the Research UNE repository (RUNE) at the following link https://aus01.safelinks.protection.outlook.com/?url=https%3A%2F%2Fhdl.handle.net%2F1959.11%2F54805&data=05%7C01%7Cgjamieso%40myune.mail.onmicrosoft.com%7C6257298be77e460f0cb608db572371ec%7C3e104c4f8ef24d1483d8bd7d3b46b8db%7C0%7C0%7C638199581972550173%7CUnknown%7CTWFpbGZsb3d8eyJWIjoiMC4wLjAwMDAiLCJQIjoiV2luMzIiLCJBTiI6Ik1haWwiLCJXVCI6Mn0%3D%7C3000%7C%7C%7C&sdata=B3UkQRqoqGIXkA%2Fm5%2Ba76k30rNYuu0Xg8lMP7b%2Bk2r0%3D&reserved=0.

**TABLE 1:** Happy and fearful faces for each trial type.

Trial type	Fearful	Happy
C	24	34
I	49	45
CI	23	20
II	25	26

C, congruent trial; I, incongruent trial; CI, incongruent trial preceded by congruent trial; II, incongruent trial preceded by incongruent trial.

**FIGURE 3:**
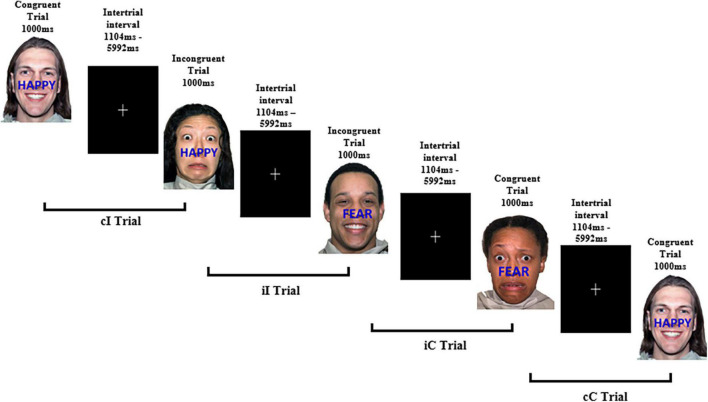
Example experimental paradigm of the affective face-word Stroop task. Examples of congruent and incongruent trials to examine affective conflict as well as trial combinations for conflict adaptation effects. cI, incongruent trial preceded by a congruent trial; iI, incongruent trial preceded by an incongruent trial; iC, congruent trial preceded by an incongruent trial; cC, congruent trial preceded by a congruent trial. Face stimuli sourced from the NimStim set of Facial Expressions (Tottenham et al., 2009) and available to the scientific community at https://macbrain.org/resources/. All faces are from the Nim Stim data set publicly available for scientific research.

The task consisted of the presentation of 154 images across two blocks of trials, separated by a break. The first block comprised of 78 trials and the second block of 76 trials. The first trial of each block is not assigned a stimulus sequence. Stimuli were presented for 1000 ms and were separated using an interstimulus interval (ISI) that jittered between 1104 and 5992 ms to decrease expectancy effects. These ISI are longer than typical in an ERP paradigm and were chosen to match those reported by Etkin et al. (2006) in their original fMRI study. A white central fixation cross appeared on a black background for the duration of the ISI (refer Figure 3 for details). The trial sequence for the two blocks was programmed with MATLAB and presented in a pseudo-random order to ensure the same trial type did not appear consecutively to avoid repetition priming effects (Mayr et al., 2003). A breakdown of stimulus characteristics is listed in Supplementary Table 1.

### 2.3. Electroencephalogram data acquisition

**FIGURE 4:**
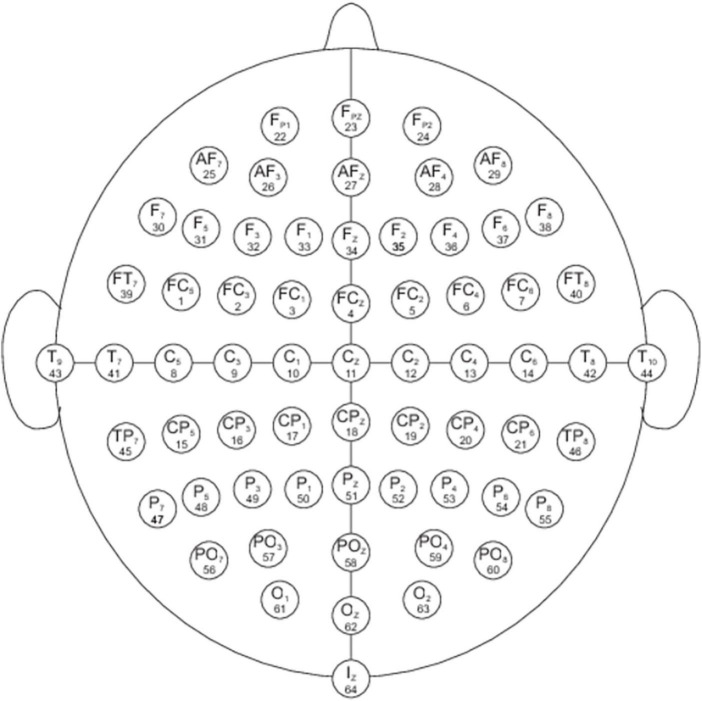
Electrode locations of International 10-20 system for EEG recording for the 64 channel Neuroscan Quik-Cap. https://compumedicsneuroscan.com/wp-content/uploads/quik-cap-64ch.jpg. All faces are from the Nim Stim data set publicly available for scientific research.

Electroencephalogram was recorded using a 64-channel (channels M1 and M2 were not used in the recording) Neuroscan QuikCap (Compumedics USA Ltd., El Paso, TX, USA) arranged in accordance with the international 10/20 system and aligned with the anatomical nasion and inion points (see Figure 4). Electrodes were composed of Ag/AgCl. Signals were acquired and digitized using a SynAmps RT 24-bit digital amplifier (Compumedics USA Ltd., El Paso, TX, USA) at a sampling rate of 500 Hz and passed through a bandpass filter of DC to 200 Hz. The amplifier was connected to Curry 7 Acquisition software (Compumedics USA Ltd., El Paso, TX, USA) located on a Dell T5700 laptop, while the STIM2 software for the emotional Stroop task was run on a second Dell T5700 laptop. The recording reference was an electrode midpoint between electrodes Cz and CPz. Recordings were converted to a common average reference offline.

### 2.4. Procedure

After providing written informed consent, participants completed a demographic information form and all cap electrodes were prepared using an electrolyte solution administered with an electronic pipette into the QuikCell sponge of the electrodes. Impedance levels were below 10 kΩ at the commencement of recording.

Participants were instructed to relax into a comfortable position approximately 1 m from the computer screen, and to remain as quiet and still as possible for the duration of the experiment. All participants commenced EEG recordings with 2 min of eyes closed rest followed by 2 min of eyes open rest. EEG recording was paused while participants were provided with instructions for the Stroop task. Participants were instructed to respond to the emotional word and not the facial expression as quickly and as accurately as possible and to avoid missing a response. They were instructed to nominate their response by using their right hand only on the numeric keyboard, pressing number 1 to indicate the word “FEAR” or number 3 to indicate the word “HAPPY.” In order to counterbalance the response type with motor preparation, these numbers were reversed for each alternate participant. The break allowed between the two experimental blocks was up to 20 s.

Recording recommenced at the completion of the verbal instructions immediately prior to commencement of the first block of the emotional Stroop task. Testing took approximately 90 min per participant per session, including equipment set up.

### 2.5. EEG analysis

Data processing was conducted using MATLAB r2018a and IBM SPSS Statistics v27. EEG was re-referenced offline to a common average channel. A 2nd order Butterworth bandpass filter set at 0.5–48 Hz was then applied. Raw data was first visually inspected to reject any obvious segments of artifact removed from the EEG. Following this, independent components analysis (Infomax algorithm) was conducted via *Fully Automated Statistical Thresholding for EEG artifact Rejection* (Nolan et al., 2010) to reject systematic artifacts in the dataset caused by biological processes (e.g., eye movement, scalp tension, and breathing) and environmental and electromagnetic interference (e.g., mains electric fields or activity from mobile phones).

Event Related Potentials (ERPs) are a measure of cortical activity and are time and phase locked to a stimulus. EEG waveforms were epoched between 250 ms pre-stimulus and 500 ms post-stimulus. EEGLAB version 14.1.1 (Delorme and Makeig, 2004) was used for criterion-based artifact rejection. Criteria were set to reject epochs with amplitudes greater than 60 μV, as these were likely to contain non-cortical signals. The data were visually inspected for any remaining epochs containing atypical artifact and manually removed.

### 2.6. EEG source analysis

Cortical source localization analyses of ERPs during each condition were conducted using The Key Institute eLORETA (exact low resolution brain electromagnetic tomography; Pascual-Marqui et al., 2011) package, which provides a single weighted minimum norm solution to the inverse problem, resulting in zero error localization in the gray matter (Pascual-Marqui, 2002; Marco-Pallarés et al., 2005). Cortical sources of task related activity were identified by computing three-dimensional distribution of current source density [the description of the method is detailed in Pascual-Marqui (2009) and Pascual-Marqui et al. (2011)] in 6239.5 mm^3^ gray matter voxels throughout the cortex. The weights utilized by eLORETA yield images of current density in a standardized realistic head model (Fuchs et al., 2002) based on the MNI152 template (Mazziotta et al., 2001).

### 2.7. Statistics

Mean reaction times for correct responses were calculated for each individual in each of the I, C, CI, and II trial types. RTs faster than 200 ms and slower than 1050 ms were excluded from the mean RT calculation. Mean RT and response accuracy rate differences between trial types were analyzed in SPSS v27 using paired samples *t*-tests and the Wilcoxon signed rank test, respectively. The eLORETA software package was used to conduct statistical testing of differences in voxel activity between conditions. We first computed paired samples *t*-tests at each voxel between designated conditions. To control for the false discovery rate we next employed the exceedance proportion test [see Friston et al. (1990, 1991) for details], a method adapted from neuroimaging to determine thresholds for a group of voxels above which the set of voxels as a whole has the designated *p*-value. The exceedance proportion test in the eLORETA software package calculates a series of progressively increasing thresholds (set at deciles between the lowest and highest obtained voxel statistic) and the associated probabilities. In each case, when the maximum voxel statistic was significant [as determined by non-parametric randomization testing; see Nichols and Holmes (2002)] we applied the threshold immediately below that threshold containing only the maximum voxel value and report only that set of voxels. In that case, reported Cohen’s *d* values are based upon the threshold *t* value.

## 3. Results

### 3.1. Behavioral data

Data obtained were analyzed using reaction times and error rates (Table 2). The assumptions of normality were met for all variables except those related to accuracy; for those analysis the equivalent non-parametric tests were used.

**TABLE 2:** Reaction times and error rates for experimental conditions across sessions.

	Stroop		Gratton	
	**C**	**I**	**CI**	**II**
RT (ms)	577.84 (75.6)	585.56 (75.7)	583.24 (75.8)	591.25 (76.6)
Accuracy (%)	95.14 (4.97)	95.08 (5.42)	95.09 (5.57)	95.08 (6.00)

Standard deviations are presented in parentheses. RT, reaction time in milliseconds.

#### 3.1.1. Reaction times

A paired samples *t*-test was used to compare participants’ mean reaction times on incongruent trials and congruent trials (see Table 2). There was a significant Stroop effect for trial congruency on participants’ reaction times, with slower reaction times found for incongruent trials; *t*(47) = 2.080, *p* = 0.021 (one tailed), Cohen’s *d* = 0.28.

A further paired samples *t*-test was used to compare participants’ mean reaction times on II trials and CI trials (see Table 2). There was a significant effect for prior trial congruency on participants’ reaction times to a current incongruent, with slower reaction times found for II trials than for CI trials; *t*(47) = −2.386, *p* = 0.021 (two tailed), Cohen’s *d* = −0.32. A two-tailed *p*-value is reported in this case because the direction of the significant difference is the reverse of that described by the Grattan effect.

#### 3.1.2. Accuracy data

Mean accuracy was consistently high across all trial types. A Wilcoxon signed rank test indicated that II trials had a non-significant influence on participant accuracy scores compared with CI trials; *z* = −0.062, *p* = 0.950, two-tailed, *r* = 0.009.

### 3.2. Averaged face-word affective Stroop VEPs and global field power

#### 3.2.1. Congruent and incongruent trials

**FIGURE 5:**
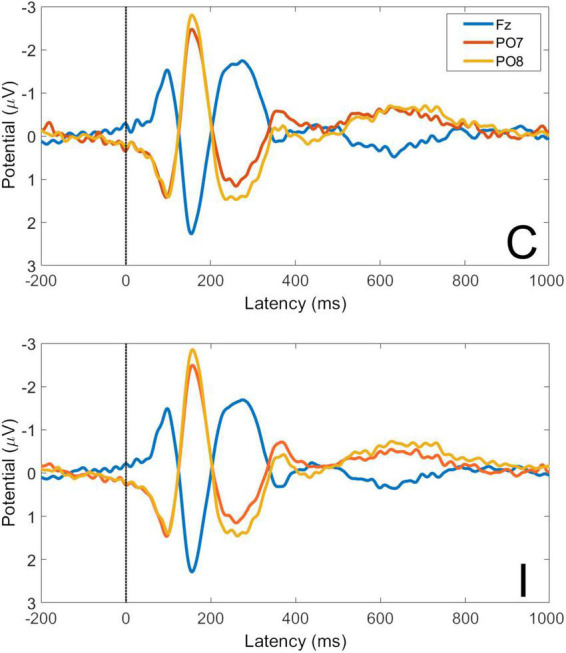
Visual evoked potentials for congruent and incongruent trials at electrodes Fz, PO7, and PO8. C, congruent trials; I, incongruent trials. All faces are from the Nim Stim data set publicly available for scientific research.

Averaged VEPs for C and I trials are shown for electrodes Fz, PO7, and PO8 in Figure 5. Waveforms show an expected topography for face VEPs with a maximum negative peak in the right lateral parietal-occipital region. The P1 peak occurs at 98 ms post-stimulus and the N170 peak at 156 ms post-stimulus.

**FIGURE 6:**
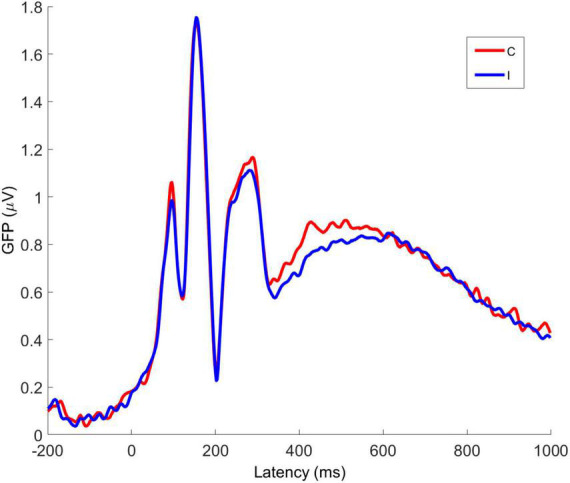
Averaged global field power for congruent and incongruent trial VEPs. C, congruent; I, incongruent. All faces are from the Nim Stim data set publicly available for scientific research.

Global Field Power (GFP) is the spatial variance of the EEG/ERP signal across all electrodes at each time point in the signal. As EEG/ERP topography is essentially the information on which source analysis operates, GFP provides an objective method by which to establish time windows for source analysis that are fitted to a particular data set but do not pre-empt the results of the analysis. The GFP time-series for C and I trials is shown in Figure 6. It can be seen that the timing of the first GFP peak closely matches that of the P1 in Figure 5, and that the timing of the second peak closely matches that of the corresponding N170 peak in the same figure.

#### 3.2.2. Congruent-incongruent and incongruent-incongruent trials

**FIGURE 7:**
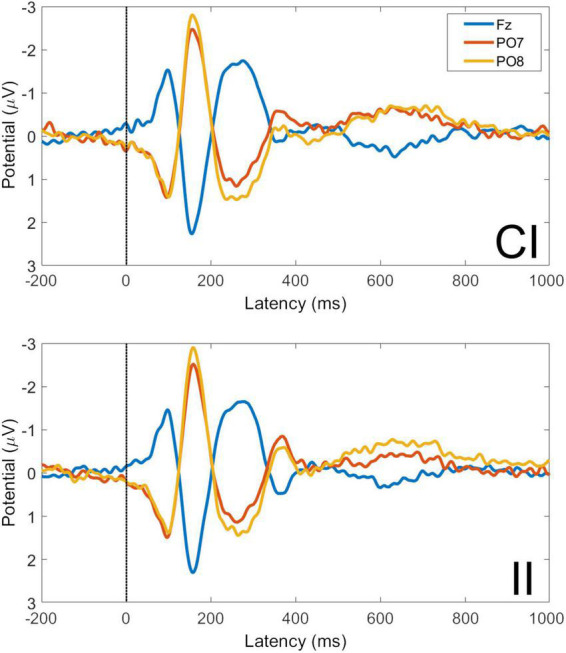
Visual evoked potentials for congruent-incongruent and incongruent-incongruent trials at electrodes Fz, PO7, and PO8. CI, congruent-incongruent trial; II, incongruent-incongruent trials. All faces are from the Nim Stim data set publicly available for scientific research.

**FIGURE 8:**
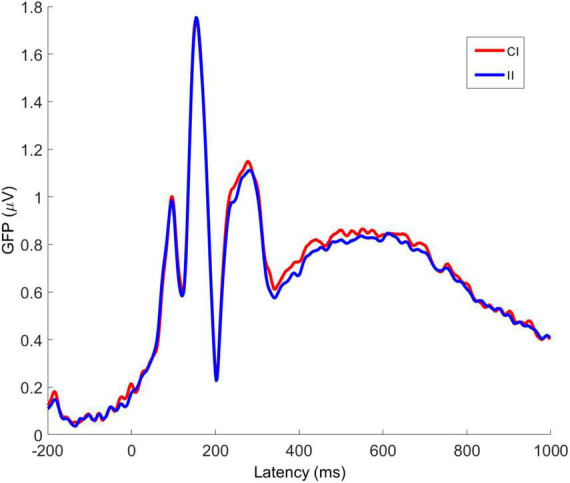
Averaged global field power for congruent-incongruent and incongruent-incongruent trial VEPs. CI, congruent-incongruent; II, incongruent-incongruent. All faces are from the Nim Stim data set publicly available for scientific research.

Averaged VEPs for CI and II trials are shown for electrodes Fz, PO7, and PO8 in Figure 7. The P1 peak occurs at 98 ms post-stimulus and the N170 peak at 156 ms post-stimulus. The GFP time-series for CI and II trials is shown in Figure 8. The timing of the first GFP peak closely matches that of the P1 in both Figure 7 and the timing of the second peak closely matches that of the corresponding N170 peak in the same figure.

### 3.3. eLORETA source analysis

Event related potential source analyses were conducted on averaged waveforms at all 62 recording electrodes. The grand averages at each electrode, in each condition, as well as individual averages and behavioral data for each participant are available for inspection and may be downloaded from the following RUNE link https://aus01.safelinks.protection.outlook.com/?url=https%3A%2F%2Fhdl.handle.net%2F1959.11%2F54805&data=05%7C01%7Cgjamieso%40myune.mail.onmicrosoft.com%7C6257298be77e460f0cb608db572371ec%7C3e104c4f8ef24d1483d8bd7d3b46b8db%7C0%7C0%7C638199581972550173%7CUnknown%7CTWFpbGZsb3d8eyJWIjoiMC4wLjAwMDAiLCJQIjoiV2luMzIiLCJBTiI6Ik1haWwiLCJXVCI6Mn0%3D%7C3000%7C%7C%7C&sdata=B3UkQRqoqGIXkA%2Fm5%2Ba76k30rNYuu0Xg8lMP7b%2Bk2r0%3D&reserved=0.

Based on the GFP data, four time windows were established for the eLORETA analysis of cortical source activity differences between CI and II trials and between C and I trials. The first window was defined by the onset of the first GFP peak until the first peak (50–98 ms post stimulus). Note that the termination of this time window closely corresponds both to the P1 peak and to the expected magnocellular activation of the amygdala. The second window was defined by interval between the first GFP peak and the following GFP minimum (98–118 ms post-stimulus). The third window is defined by period from the GFP onset of the N170 component in the VEP until the second GFP peak (118–156 ms post-stimulus). The final window corresponds to the resolution of the N170 component and is defined by the time from the second GFP peak until the second GFP minima (154–204 ms post-stimulus).

#### 3.3.1. Incongruent-incongruent vs. congruent-incongruent trials

For the time window 96–118 ms post stimulus the exceedance proportion test identified a group of 26 voxels above the threshold value of *t*(41) = −3.847 with a probability of *p* < 0.002 for the set. At the threshold value, Cohen’s *d* = 0.64 (a medium effect size).

**FIGURE 9:**
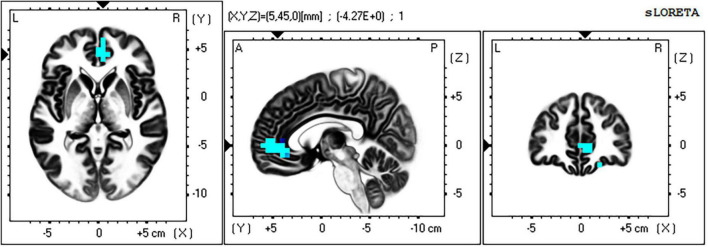
Significant voxels for incongruent-incongruent vs. congruent-incongruent trials 96–118 ms post stimulus. Blue indicates II < CI, *p* < 0.002. Max voxel MNI 5 45 0, right BA 32 (rACC). All faces are from the Nim Stim data set publicly available for scientific research.

The maximum voxel statistic (*t* = −4.27) was located at MNI (5, 45, 0) right BA32. Supra threshold voxels are shown in blue in Figure 9 in each Cartesian plane about the max voxel location.

The full list of suprathreshold voxels is presented in Supplementary Table 2. Significant voxels are right lateralized and fall within frontal regions BA32 (rACC), BA10, and BA11.

The II vs. CI contrast found no significant voxels in any other time window.

#### 3.3.2. Incongruent vs. congruent trials

For the time window 118–156 ms post stimulus, the exceedance proportion test identified a group of 20 voxels above the threshold value of *t*(41) = −3.537 with a probability of *p* < 0.003 for the set. At the threshold value, Cohen’s *d* = 0.559 (also a medium effect size).

**FIGURE 10:**
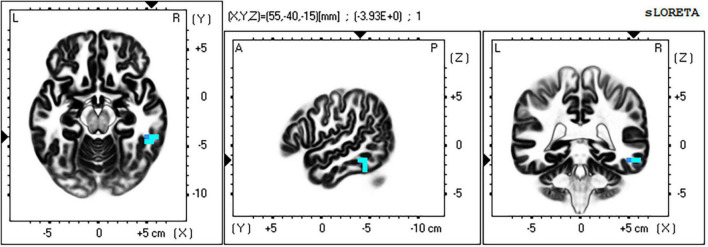
Significant voxels for incongruent vs. congruent trials 118–156 ms post stimulus. Blue indicates I < C, *p* = 0.003. Max voxel MNI 55 –40 –15, right BA 20 (FFA). All faces are from the Nim Stim data set publicly available for scientific research.

The maximum voxel statistic *t* = −3.39 was located at MNI (55, −45, 15) at right BA20. Supra threshold voxels are shown in blue in Figure 10 in each Cartesian plane about the max voxel location.

The full list of suprathreshold voxels is presented in Supplementary Table 3. Significant voxels are again right lateralized and fall mainly within middle and inferior temporal lobe, BA20 and BA 37 consistent with the FFA. Another region in this significant grouping is BA6 in the frontal cortex.

The I vs. C contrast found no significant voxels in any other time window.

## 4. Discussion

We sought to extend the understanding of how conflicts in affective signals between verbal and non-verbal channels are resolved in the brain. We utilized the temporal resolution of the EEG to explore within-trial dynamics controlling the processing of these conflicts in the affective face-word Stroop paradigm developed by Etkin et al. In doing so, we adapted the original paradigm so that participants must respond to the word (verbal) rather than the face cue. Our first concern was to establish that this paradigm would produce both Stroop interference and control type effects at the behavioral level. This was confirmed with RTs for I trials found to be significantly longer than C trials.

Next, we sought to establish the presence of a control effect at the behavioral level between II (considered to be high control) and CI (considered to be low control) trials. There was a significant difference in RT between these two conditions, however, it was the CI trials which showed faster responses than the II trials. This contradicted our expectation of a Gratton type control effect. Instead of the high control condition being faster than the low control condition, it was slower. In fact, the effect size for this difference was larger than the effect size for the Stroop effect itself.

This pattern is similar to another widely studied behavioral control effect known as the Rabbitt effect (Rabbitt, 1966) (also known as post-error slowing), in which a correct response following an error is found to be slower than a correct response following another correct response. The error response that triggers the control adjustment by post error slowing is a high conflict event (Botvinick et al., 2001). In the present case slowing on the subsequent trial is also triggered by a high conflict stimulus on the previous trial. Thus, in both cases, the cognitive control resources required to perform the current task (indexed by RT) vary as a function of prior demands on control resources. An I trial is presumed to induce greater conflict than a C trial; consequently it will require greater activation of the relevant control resources to respond to than a C trial. As a result, the comparison between an I trial and a C trial is not only one of stimulus or response conflict but also of the level of required control (fast and accurate response to I trials requires more control than to C trials). In the case of the Gratton effect, greater control (elicited by an incongruent stimulus) on a prior trial will result in a higher level of activation (or readiness for activation) of those control resources on the current trial. So the current trial they will require less resources to initiate control and/or control will be engaged more rapidly. In the case of the Gratton effect, this translates into reduced response times but in this case—as in the Rabbitt effect—enhanced control is linked to slower response times.

This could be explained if the response criteria of participants was biased toward accuracy rather than speed. Botvinick et al. (2001) provide such a model, simulating the Rabbitt effect where high conflict trials trigger a change in response criteria. Although participants were instructed that both speed and accuracy were important, this does not guarantee that this was the case. Some support for this interpretation may be seen in the mean accuracy rates reported in Table 2 above: mean accuracy was identical (to one decimal point) for all trial types, whereas in the results reported by Etkin et al. (2006) accuracy for C trials was greater than for I trials (as is typical for Stroop type paradigms). A bias toward accuracy over response time would mean that, once engaged, control resources would be active for longer before response selection to check that an error was not being made. In this study, such a motivational set may be due to our sample who were comprised of administrative workers in a government authority, and who were tested in a workplace setting. Whilst unexpected, behavioral results do support the conclusion that higher levels of stimulus conflict in prior trials recruit greater control in responses to current conflict trials, II trials continue to express greater behavioral control than in CI trials.

The comparison of cortical source activity between high control (II) and low control (CI) trials confirmed a highly significant cluster of affective-conflict control related voxels centered in the right rACC, with lower activity on the slower II trials and higher activity on the faster CI trials. Applying the logic of Botvinick et al. from brain imaging studies to these ERP source analysis results, this CI > II activation difference would be taken to indicate conflict (rather than control) related activity, whereas II > CI voxels are taken to indicate control related activation. That is, based on the reasoning that CI trials are lower in control (hence higher in conflict) than II trials because they are *slower* to respond (the Gratton effect) but in this case they are *faster* therefore following that deeper logic we continue to interpret this finding as a control response induced by affective–conflict.

This affective-conflict-stimulus evoked control response was observed only in the 98–118 ms post-stimulus time window. This time window lies in the resolution of the P1 component of the VEP and immediately prior to the onset of the N170 component (which in this case peaked at 156 ms post stimulus). This occurs very early in the stimulus processing response, prior to the completion of sensory stimulus analysis, and hence prior to response selection and motor preparation. It precedes, rather than follows response conflict within the trial, yet it is sensitive to congruency in the previous trial. It may be that it is the discrepancy between prior trial stimulus-conflict and the current trial stimulus conflict (a form of expectancy violation) that is triggering the control response in this case.

The timing of BA32 control activity in this study is too early to be triggered by either sensory analysis of stimulus conflict or response conflict. Etkin et al. (2006) reported that control related BA32 activity was positively correlated with amygdala activity in prior trials and negatively correlated with amygdala activity in the current trial. While activity in the amygdala cannot be measured in the current study due to the limitations of the scalp EEG, it is known that affective stimuli evoke a rapid amygdala response via fast links from the pulvinar nucleus of the thalamus, which occurs prior to sensory analysis in visual cortex (Garvert et al., 2014). If BA32 control activation in this paradigm is positively related to amygdala activation in the previous trial [as in Etkin et al. (2006)], then this increased activation in the amygdala is a strong candidate for triggering the control response observed here.

In the current study, control related rACC activity was found only in the right hemisphere, unlike Etkin et al. (2006) where it was found only in the left hemisphere. This is to be expected if the neural circuitry of control being exercised here is also lateralized. In our study, the conflicting affective stimuli (faces) are processed along a right hemisphere pathway that we expected to be influenced by rACC control. Whereas in the study conducted by Etkin et al. (2006), the conflicting affective information was carried by the word which is processed along a left hemisphere pathway. The lateralization of rACC affective control for affective words vs. faces, evident when both studies are viewed in conjunction, is a new finding which merits further consideration in relation to the development of other lateralized models of affective regulation (Craig, 2011; Bruder et al., 2017).

In addition to the specific predictions made concerning rACC activation in relation to affective control, there were further unanticipated findings in our contrast of II and CI trials in this time window. While the locus of significant voxels was clearly right rACC (BA32), fully half of the significant group of voxels identified lay in right orbitofrontal cortex (BA10 and BA11; see Supplementary Table 2). Tracing studies show that rACC is a rich hub connecting many regions across the frontal cortex (Tang et al., 2019) including BA10 and BA11. These results suggest that the control related effects of rACC on the amygdala likely include interactions with other frontal regions, which may provide a basis for integrating cognitive processes with affective-conflict regulation.

The contrast between I and C trial types revealed a highly significant group of voxels mostly in right mid-inferior temporal cortex (including BA 37 fusiform gyrus; see Supplementary Table 3) and the right FFA, that was less active in the I condition than in the C condition in the time window 118–156 ms post-stimulus. Thus, in the time window between onset and peak of the N170 component, activity near right FFA is reduced at the time when affective information is being identified in the incongruent face stimulus. This is the same contrast in which Etkin et al. (2006) identified amygdala activation in response to conflict (I > C) in the affective face-word stimuli.

The amygdalae have reciprocal connections with extrastriate visual processing areas in inferior and mid temporal regions, so the interaction of these regions may occur throughout the response to each AFWS trial. Current literature indicates that the initial response of the amygdalae to affectively salient stimuli is initiated by fast magnocellular pathways (not accessible in the current study). Amongst many effects this early amygdala response is expected to prime extrastriate visual cortex for processing salient stimulus features, thus enhancing later processing in these regions. From the findings of Etkin et al. (2006), in the case of affective conflict (AFWS), this would then trigger control related activity in rACC; in that study left rACC in the case of face target, and in the current study right rACC in the case of word target. This would then result in down regulation of the amygdala with subsequent down regulation of affective-face processing in right FFA. We found reduced right inferior temporal and fusiform gyrus activity in the I trials which followed immediately from the time widow in which right rACC (BA32) control related activation was identified, consistent with the predictions of our model presented previously in Figure 1.

Current findings in the I vs. C contrast correspond closely with the ERP results reported by Zhu et al. (2010) for the word target version of the AFWS. They report a reduction in the N170 component maximal at right lateral posterior recording sites, and likely identify similar cortical source activity (not analyzed in that study) to that underlying those results. Note that while the inference drawn from I > C activation in stimulus-response conflict paradigms in the brain imaging literature (Botvinick et al., 1999; Egner and Hirsch, 2005b) dictates that this is interpreted as conflict related in the case of ERP and ERP source localization this functional inference is now drawn from I < C results. This indicates the need for great care in triangulating between EEG/ERP and brain imaging methods in the study of psychological phenomena. Zhu and colleagues also report the reverse finding for the face target version of the AFWS—an increase in the amplitude of the N170 component maximal at right lateral posterior recording sites for I compared to C trials. This result, considered with those of the current study, suggest increased affective-face processing of incongruent stimuli in the region of the right FFA when face is the target. A similar finding was also reported using fMRI by Egner and Hirsch (2005b) for their initial (cognitive) face-word Stroop paradigm, in which task relevant face processing in FFA was amplified in high control conflict trials. The I > C findings of Zhu et al. (2010) for face target likely indicate top-down upregulation of affective face processing. Returning to our current result, this would imply that top-down facilitation of extrastriate affective-face processing (which we suggest is driven by the salience response of the amygdala) is reduced on incongruent (high processing conflict) trials for the word target in the AFWS task. We take this to indicate that aforesaid facilitation is itself down regulated by the affective-conflict control mechanisms evoked by the stimulus incongruence.

### 4.1. Limitations

There are two important ways in which the experimental paradigm of the current study differ from Etkin et al. (2006), and both place important limitations on the interpretation of results. Firstly, the current study utilized a word target AFWS paradigm while Etkin et al. employed a face target AFWS paradigm. In order to further clarify the meaning of the current ERP source localization findings in relation to the fMRI findings of Etkin et al., it will be necessary to conduct a study which employs both face and word target versions of the AFWS in a single study [as conducted by Zhu et al. (2010)] but utilizing source localization and control-related contrasts (II vs. CI) which were not employed in that study. In addition to replication, it will be important to establish whether BA32 control related activation occurs ipsilateral to conflicting sensory processing pathways.

Secondly, the ratio of I to C trials in this study was approximately 2:1 whereas in Etkin et al. (2006) it was 1:1. The ratio of incongruent to congruent trials is known to influence the Stroop interference effect, with a higher proportion of I trials associated with reduced Stroop interference effect in RT measures (Braem et al., 2019). It is unknown how this trial type frequency effect may influence other conflict related control effects such as the Gratton effect [employed by Etkin et al. (2006)] or Rabbitt type effects (as in the current study). It is likely to enhance control (and reduce conflict) on incongruent trials and thereby diminish the Gratton effect (which was absent in the current study). It will be important to conduct an AFWS study (preferably employing both face and word response instructions) which manipulates trial type frequency across high, balanced, and low ratios of I to C trials to determine the impact on trial sequence (II, CI, IC, CC) reaction times.

It may also be useful for researchers to examine the differences in control elicited when participants are instructed on the importance of accurate responses, speedy responses, and when they receive balanced instructions. In order to unambiguously interpret I vs. C source localization differences as evidence for facilitation, absence of facilitation or conflict interference, it is necessary to include affectively neutral face and word stimuli in the AFWS paradigm [as employed for example by Ovaysikia et al. (2011) in their related fMRI study]. Confirmation of the role of the amygdala proposed in the current model would require future studies to employ magnetoencephalogram (MEG) recording, which is able track both cortical and amygdala activity with the temporal of the EEG (Dumas et al., 2013).

## 5. Conclusion

This research compliments and extends the findings of Etkin et al. (2006) on the regulation of affective-conflicts in sensory information, in particular between words and faces using an adaptation of the affective face-word Stroop paradigm. Using contrasts derived from cognitive neuroscience research on Stroop type tasks, they identified right amygdala activation as a primary emotional conflict response in the face response AFWS task, and left rACC as a source of affective-control which directly inhibits amygdala activation. By leveraging the precise temporal resolution of the EEG, we were able to obtain within-trial timings for initiation of rACC affective-control activation, and for the subsequent effects (plausibly expressions of control) on the extrastriate visual pathways which processed the conflicting affective-face signals.

At a behavioral level, we were able to identify a novel Rabbitt type control effect. The Rabbitt effect has been shown to be modulated by the same conflict related activity (in this case dorsal rather than rostral ACC) as the Gratton effect in a cognitive Stroop color naming task (Kerns et al., 2004). These results indicate that the control response to affective conflict can toggle between Rabbitt-type and Gratton effects, although the precise circumstances which trigger this switch and the underlying mechanism remain to be identified.

We have confirmed the role of rACC in the control response to affective conflict and identified that these effects are lateralized. Considering these findings with those of Etkin et al. (2006), it appears that right rACC is engaged in regulating affective processing when conflict arises from right hemisphere processing, and left rACC in regulating conflict arising from left hemisphere processing pathways. Our results indicate that the initiation of rACC control involves evoked cortical potentials that are not registered in the results available through fMRI. This highlights the importance of convergent (not merely parallel) inquiry in this field, and the differences in interpretation required when dealing with EEG/ERP and BOLD fMRI results.

Current results support the view that rACC regulation of affective-conflicts are triggered within trials by early conflict-related amygdala activation, leading to down regulation of the amygdala response and hence to down regulation of the amygdala’s attentional facilitation of selected affective signals (in this case targeting the right FFA). While the current findings extend our understanding of how the brain responds to affective conflicts, the ultimate utility of such knowledge lies in understanding the real world of complex social interactions in which the management of affective conflicts plays a key role in negotiating the complex interactions between self and others.

## Data availability statement

The raw data supporting the conclusions of this article will be made available by the authors, without undue reservation.

## Ethics statement

The studies involving human participants were reviewed and approved by Human Research Ethics Committee. The patients/participants provided their written informed consent to participate in this study.

## Author contributions

GJ contributed to planning, writing, and analysis. JP contributed to planning, writing, analysis, and data collection. IE contributed to writing and analysis. AH contributed to planning and writing. All authors contributed to the article and approved the submitted version.
