# Supplementary material for: Conflict and control in cortical responses to inconsistent emotional signals in a face-word Stroop
Source: Front Hum Neurosci. 2023 Jun 30;17:955171. doi: 10.3389/fnhum.2023.955171 (PMC10349396; doi:10.3389/fnhum.2023.955171)
Supplement: Supplementary file 1 [file Data_Sheet_1.docx]

Supplementary Material

**Supplementary Table S1**. Stroop Stimuli Details

| **Face-word Combination** | **Number of photos of this combination** |
| --- | --- |
| Female fear ‘FEAR’ (word in capitals refers to the word across the face) | 5 |
| Female fear ‘HAPPY’ | 11 |
| Female happy ‘FEAR’ | 9 |
| Female happy ‘HAPPY; | 7 |
| **TOTAL FEMALE FACE WORD COMBINATIONS THAT APPEARED** | **32** |
| Male fear ‘FEAR’ | 5 |
| Male fear ‘HAPPY’ | 14 |
| Male happy ‘FEAR’ | 14 |
| Male happy ‘HAPPY’ | 8 |
| **TOTAL MALE FACE WORD COMBINATIONS THAT APPEARED** | **41** |
| **TOTAL CONGRUENT STIMULI** | **59** |
| **TOTAL INCONGRUENT STIMULI** | **95** |

| **Different female faces of racial type** | | |
| --- | --- | --- |
| Caucasian | Brunette | 4 |
|  | Blonde | 1 |
|  | Ginger | 2 |
| African American |  | 3 |
| Asian |  | 3 |
| **TOTAL DIFFERENT FEMALE FACES OF RACIAL TYPE** | | **13** |

| **How many times the female faces above appeared in the face word Stroop task** | | |
| --- | --- | --- |
| Caucasian | Brunette | 6 |
|  | Blonde | 4 |
|  | Ginger | 5 |
| African American |  | 9 |
| Asian |  | 8 |
| **TOTAL NUMBER OF FEMALE FACE WORD COMBINATIONS THAT APPEARED** | | **32** |

| **Different male faces of racial type** | |  |
| --- | --- | --- |
| Caucasian | Brown hair | 5 |
|  | Blonde | 5 |
|  | Ginger | 2 |
| African American |  | 6 |
| (No Asian faces) |  | 0 |
| **TOTAL DIFFERENCE MALE FACES OF RACIAL TYPE** | | **18** |

| **How many times the male faces above appeared in the face word Stroop Task** | |  |
| --- | --- | --- |
| Caucasian | Brown hair | 11 |
|  | Blonde | 7 |
|  | Ginger | 10 |
| African American |  | 13 |
| **TOTAL NUMBER OF MALE FACE WORD COMBINATIONS THAT APPEARED** | | **41** |

**Supplementary Table S.2**

Significant voxels II versus CI 96-118 ms

| X(MNI) | Y(MNI) | Z(MNI) | *t* | BA |
| --- | --- | --- | --- | --- |
| 5 | 45 | 0 | -4.27 | 32 |
| 5 | 50 | 0 | -4.23 | 32 |
| 5 | 45 | -5 | -4.15 | 32 |
| 5 | 40 | 0 | -4.14 | 32 |
| 5 | 40 | -5 | -4.13 | 32 |
| 5 | 55 | 0 | -4.08 | 10 |
| 5 | 50 | 5 | -4.07 | 10 |
| 10 | 45 | -5 | -4.06 | 32 |
| 0 | 50 | 0 | -4.06 | 32 |
| 5 | 50 | -5 | -4.04 | 10 |
| 10 | 40 | -10 | -4.02 | 10 |
| 0 | 45 | 0 | -4.02 | 32 |
| 10 | 40 | -5 | -4.01 | 10 |
| 5 | 55 | 5 | -3.99 | 10 |
| 0 | 60 | 10 | -3.99 | 10 |
| 20 | 45 | -20 | -3.98 | 11 |
| 5 | 35 | -5 | -3.92 | 32 |
| 10 | 50 | -5 | -3.92 | 10 |
| 10 | 45 | 0 | -3.91 | 32 |
| 0 | 55 | 10 | -3.89 | 10 |
| 20 | 40 | -20 | -3.88 | 11 |
| 5 | 40 | -10 | -3.88 | 32 |
| 5 | 60 | 0 | -3.87 | 10 |
| 15 | 50 | -15 | -3.86 | 11 |
| 10 | 35 | -10 | -3.85 | 32 |
| 5 | 55 | -5 | -3.85 | 10 |

**Supplementary Table S.3.**

Significant voxels I versus C 118 – 156 ms

| X(MNI) | Y(MNI) | Z(MNI) | *t* | BA |
| --- | --- | --- | --- | --- |
| 55 | -40 | -15 | -3.93 | 20 |
| 55 | -45 | -20 | -3.86 | 37 |
| 50 | -45 | -20 | -3.84 | 37 |
| 55 | -45 | -25 | -3.82 | 37 |
| 50 | -35 | 0 | -3.79 | 22 |
| 50 | -45 | -15 | -3.77 | 37 |
| 55 | -45 | -15 | -3.71 | 20 |
| 15 | 5 | 60 | -3.70 | 6 |
| 15 | 10 | 65 | -3.70 | 6 |
| 15 | 5 | 55 | -3.69 | 6 |
| 50 | -45 | -25 | -3.65 | 37 |
| 15 | 10 | 55 | -3.64 | 6 |
| 60 | -45 | -20 | -3.64 | 20 |
| 15 | 5 | 65 | -3.64 | 6 |
| 15 | 5 | 50 | -3.60 | 24 |
| 30 | -25 | -25 | -3.59 | 35 |
| 30 | -25 | -30 | -3.58 | 36 |
| 45 | -45 | -20 | -3.57 | 37 |
| 60 | -40 | -15 | -3.56 | 21 |
| 25 | -25 | -20 | -3.55 | 35 |
